# TACC3 is a prognostic biomarker for kidney renal clear cell carcinoma and correlates with immune cell infiltration and T cell exhaustion

**DOI:** 10.18632/aging.202668

**Published:** 2021-03-10

**Authors:** Xiaoyan Fan, Boyi Liu, Zhiyu Wang, Dongwei He

**Affiliations:** 1 Department of Oncology, Hebei General Hospital, Shijiazhuang, Hebei, P.R. China; 2 Department of Neurobiology and Acupuncture Research, The Third Clinical Medical College, Zhejiang Chinese Medical University, Key Laboratory of Acupuncture and Neurology of Zhejiang Province, Hangzhou, Zhejiang, P.R. China; 3 Department of Immune-Oncology, The Fourth Hospital of Hebei Medical University, Shijiazhuang, Hebei, P.R. China; 4 Laboratory of Pathology, Hebei Cancer Institute, The Fourth Hospital of Hebei Medical University, Shijiazhuang, Hebei, P.R. China

**Keywords:** TACC3, immune infiltration, T cell exhaustion, prognosis, kidney renal clear cell carcinoma (KIRC)

## Abstract

Growing evidence has demonstrated that transforming acidic coiled-coil protein 3 (TACC3), a member of the TACC family, may be involved in regulating cell mitosis, transcription, and tumorigenesis. However, the role of TACC3 in kidney renal clear cell carcinoma (KIRC) remains unknown. In this study, multiple databases were used to determine the pattern of TACC3 in KIRC. We found that high TACC3 expression was associated with poor overall survival (OS) in stage I, II, IV and grade 3 KIRC patients. Univariate and multivariate Cox regression analyses showed that TACC3 was an independent risk factor for OS among KIRC patients. Moreover, TACC3 expression correlated with immune cell infiltration levels of B cells, T cells (CD8+, CD4+, follicular helper, regulatory and gamma delta), total and resting natural killer cells, total and activated dendritic cells, and resting mast cells. Furthermore, T cell exhaustion markers, such as PD1, CTLA4, LAG3 and TIM-3 were highly expressed in TACC3 overexpressing tissues. In addition, GSEA analysis revealed that the role of TACC3 in KIRC may be closely linked to immune-associated pathways. Therefore, our study reveals that TACC3 is a prognostic biomarker for OS among KIRC patients and may be associated with immune cell infiltration and T cell exhaustion.

## INTRODUCTION

Kidney renal clear cell carcinoma (KIRC) represents the most common malignant epithelial neoplasm of the kidney and is one of the most common types of cancer world-wide [1]. Because KIRC is insensitive to conventional radiotherapy and chemotherapies, treatment of this disease largely relies on targeted therapy and immunotherapy. Targeted therapies include tyrosine kinase inhibitors such as sunitinib and sorafenib. However, tumor heterogeneity, dynamic variation and adaptation to treatment (such as alterations in cell death-associated signaling pathways) significantly limit the application of targeted drugs in the treatment of KIRC [2–4]. Immunotherapy, such as targeted PD-1 inhibition, can stimulate intrinsic T cells to attack tumor cells and this strategy is believed to have promising therapeutic potential for the treatment of KIRC. Encouragingly, clinical trials for advanced renal cell carcinoma have shown that patients treated with nivolumab plus ipilimumab display higher OS and objective response rates (ORR), when compared with those treated with sunitinib [5, 6]. Therefore, exploring the innate mechanism of KIRC to develop novel therapeutic strategies is vitally important for improving the clinical management of this disease.

The TACC family genes, which include TACC1, TACC2 and TACC3, were first discovered in genomic regions that are amplified in breast tumors and multiple myeloma [7]. In addition to regulating centrosomal integrity and stabilizing centrosomal microtubule nucleation during mitosis [8], TACC members have been implicated in tumorigenesis. Recently, TACC2 was reported to be involved in the androgen-mediated growth of prostate cancer [9] and was identified as a potent prognostic predictor in breast carcinoma and hepatocellular carcinoma [10, 11]. Abnormal expression of TACC1 and/or TACC3 is associated with ovarian, breast, melanoma, bladder, non-small-cell lung and prostate cancers [12–17]. Moreover, fibroblast growth factor receptor (FGFR)1-TACC1 and FGFR3-TACC3 gene fusions have been reported in various types of cancer [18, 19]. These fusion proteins are associated with aneuploidy, and display oncogenic activity. These findings suggest that TACC3 could potentially contribute to tumorigenesis.

Increasing evidence shows that overexpression of TACC3 is associated with tumor aggressiveness and poor prognosis in prostate [13], breast [12], colorectal [14], and gastric cancer [15]. More recently, Guo et al. have reported that TACC3 knockdown inhibits the proliferation and invasion of human renal cell carcinoma cell lines [20]. However, the mechanisms underlying TACC3 overexpression in KIRC still remain unclear. In the present study, we used public databases to analyze the correlation between TACC3 expression and the patient prognosis in KIRC. In addition, we proceeded to study the correlation between TACC3 expression and the tumor immune infiltration and T cell exhaustion in KIRC. Our results suggest that TACC3 may serve as a potential biomarker of prognosis in KIRC and the possible mechanism may be associated with immune cell infiltration and T cell exhaustion.

## RESULTS

### TACC3 expression in KIRC

**Figure 1:**
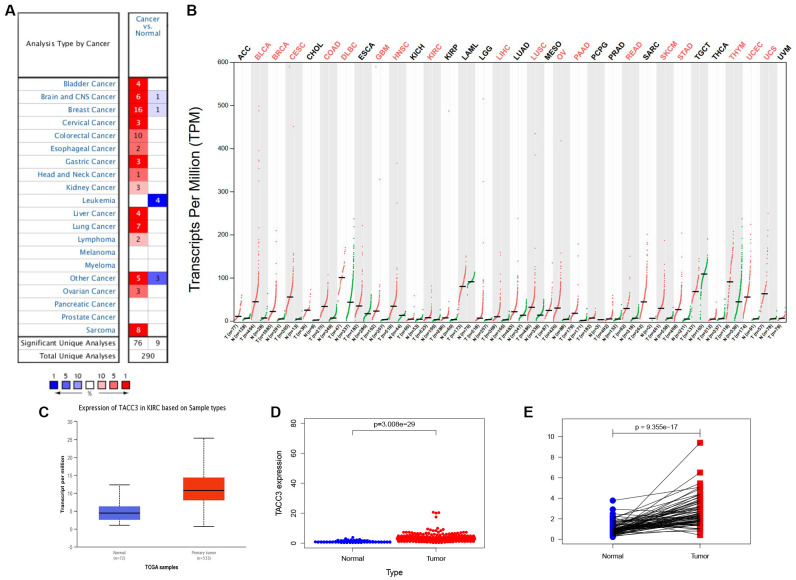
**TACC3 expression in KIRC using online databases.** (**A**) Oncomine database. Cell color is determined by the best gene rank percentile for the analyses within the cell. (**B**) GEPIA database. Significant differences were shown in red. (**C**) UALCAN database. p<0.0001. (**D**) TCGA database using R script. p=3.008e-29. (**E**) TACC3 expression in a paired comparison of KIRC and their adjacent normal tissues. Data were extracted from TCGA database. p=9.355e-17.

We first assessed the relationship between TACC3 overexpression and patient prognosis in various types of cancers. As shown in Supplementary Table 1, overexpression of TACC3 is associated with poor prognosis only in KIRC, liver hepatocellular carcinoma and thymoma. Next, the Oncomine, GEPIA and UALCAN online databases were used to determine TACC3 mRNA expression levels in cancerous and normal tissues. All of the three databases indicated that higher levels of TACC3 expression in KIRC tissues, when compared with normal tissues (Figure 1A–1C).

To further evaluate TACC3 expression in KIRC, we downloaded RNA-seq data for KIRC from TCGA and analyzed the expression of TACC3 using R software. TACC3 expression was significantly higher in KIRC tissues, when compared with normal tissues (Figure 1D, p = 3.008e-29). In addition, a pair-wise comparison of KIRC tissues and adjacent matched normal tissues revealed significantly higher TACC3 expression in the former (Figure 1E, p = 9.355e-17). These results suggest that TACC3 expression is elevated in KIRC tissues, when compared with normal tissues.

### Prognostic potential of TACC3 in KIRC

**Figure 2:**
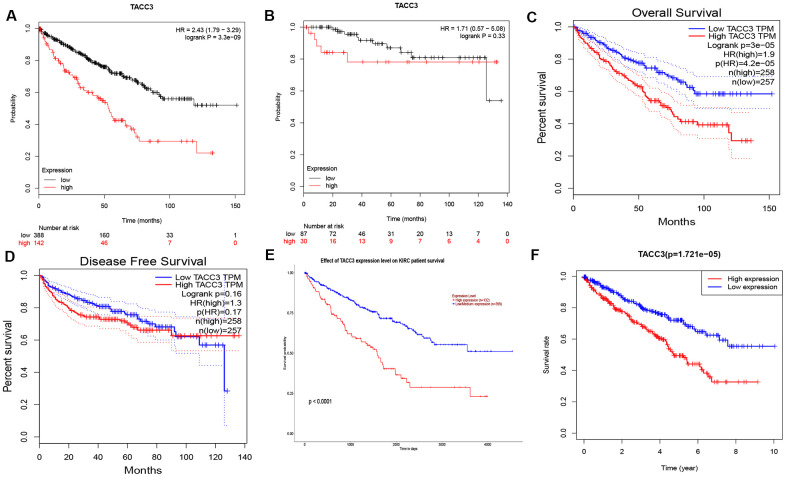
**Prognostic Potential of TACC3 in KIRC.** Various online tools were used to evaluate the relationships between TACC3 expression and patient survival. (**A**, **B**) OS and RFS in Kaplan-Meier Plotter database. (**C**, **D**) OS and DFS in GEPIA database. (**E**) OS in UALCAN database. (**F**) OS in TCGA database.

To determine the potential prognostic significance of TACC3 expression in KIRC, the Kaplan-Meier Plotter, GEPIA, and UALCAN online tools were used to evaluate the relationships between TACC3 expression and patient survival. No significant association was found between TACC3 expression and relapse-free survival (RFS) by Kaplan-Meier Plotter analysis (logrank p = 0.33, HR = 1.71, Figure 2B), or TACC3 expression and disease-free survival (DFS) by GEPIA (logrank p = 0.16, HR = 1.3, Figure 2D). However, high TACC3 mRNA expression was significantly associated with poor OS in KIRC patients in the Kaplan-Meier Plotter (logrank p = 3.3e-09, HR = 2.43; Figure 2A), GEPIA (logrank p = 3e-5, HR = 1.9, Figure 2C), and UALCAN (p < 0.0001, Figure 2E) analyses. Clinical data for KIRC were downloaded from TCGA, and the OS subsequently analyzed by R. Notably, high TACC3 expression was significantly negatively associated with the survival of patients with KIRC (p = 1.721e-05, Figure 2F). These results indicate that high TACC3 expression has a significant impact on the survival of KIRC patients.

### The role of TACC3 expression in the clinical characteristics of KIRC

To further evaluate the contribution of TACC3 to the clinical characteristics of KIRC, the relationship between TACC3 expression and clinicopathological characteristics was investigated using the Kaplan-Meier plotter tool. As shown in Table 1, TACC3 expression was negatively associated with OS in female and male patients (p = 0.00073, p = 3.7e-07, respectively), as well as patients of white race (p = 9.1e-09). Higher TACC3 expression correlated with worse OS among stage I, II, IV, and grade 3 patients (p = 0.019, p = 0.0051, p = 0.0037 and p = 0.00033, respectively). In addition, the same relationship was observed among patients with high- or low-mutation burden disease (p = 0.029 and p = 4.9e-06, respectively). However, TACC3 expression was only observed to correlate with RFS in female and low-mutation burden KIRC patients (p = 0.023 and p = 0.0036, respectively). These results suggest that high TACC3 expression significantly affects the OS of KIRC patients with stage I, II, IV and grade 3 patients.

**Table 1:** Correlation of TACC3 expression and clinical prognosis in KRIC with different clinical characteristics by Kaplan-Meier plotter.

**Clinical characteristics**	**OS**		**RFS**	
	**HR (95%CI)**	**logrank P**	**HR (95%CI)**	**logrank P**
Gender				
Female	2.31(1.4-3.8)	0.00073***	0	0.023*
Male	2.58(1.76-3.76)	3.7e-07***	2.95(0.9-9.73)	0.062
Race				
White	2.45(1.79-3.37)	9.1e-09***	1.78(0.39-8.13)	0.45
black/African American	2.83(0.6-13.27)	0.17	0	0.34
Stage				
1	2.01(1.11-3.65)	0.019*	4.21(0.59-30.06)	0.12
2	10.43(1.35-80.35)	0.0051**	-	-
3	0.75(0.42-1.33)	0.32	0.19(0.02-1.56)	0.086
4	2.41(1.31-4.45)	0.0037**	-	-
Grade				
1	-	-	-	-
2	0.75(0.42-1.36)	0.35	-	-
3	2.29(1.44-3.65)	0.00033***	0.19(0.02-1.56)	0.086
4	1.82(0.91-3.63)	0.083	-	-
Mutation burden				
High	1.83(1.06-3.19)	0.029*	0.35(0.04-2.91)	0.31
Low	5.41(2.41-12.14)	4.9e-06***	14.8(1.44-151.96)	0.0036**

- Values indicate the sample number too low for meaningful analysis.

* Values indicate p<0.05, ** values indicate p<0.01, *** values indicate p<0.001.

### High TACC3 expression is an independent risk factor for OS among KIRC patients

**Figure 3:**
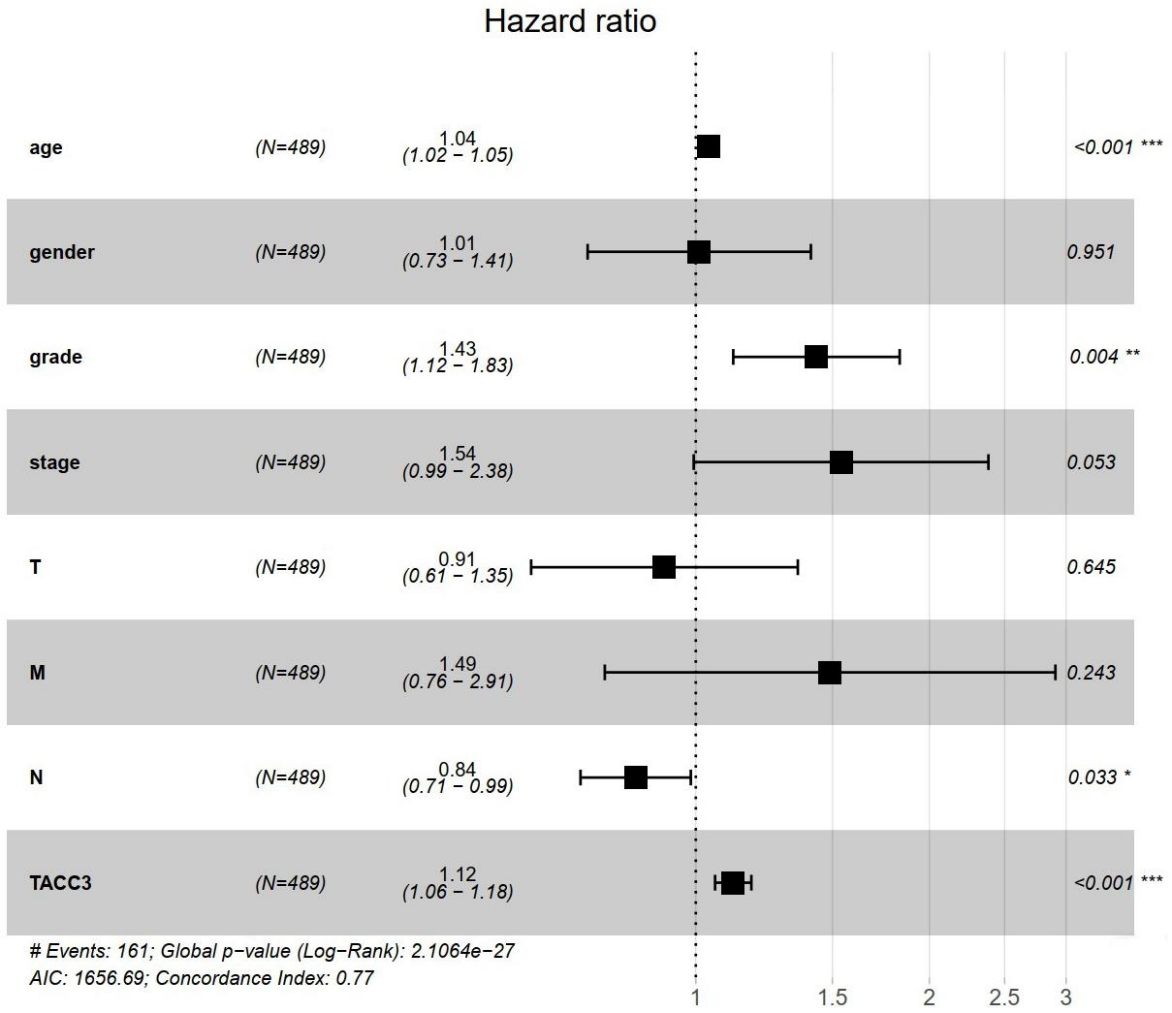
**TACC3 is an independent risk factor for OS among KIRC patients using multivariate Cox analysis.** *p<0.05, **p<0.01, ***p<0.001.

To understand whether TACC is an independent risk factor for OS in KIRC patients, univariate and multivariate Cox analyses were performed using an R script. In the univariate Cox analysis, age, grade, tumor stage, T classification, M classification, and TACC3 expression were all independent risk factors for OS (p = 2.29e-06, 9.48e-15, 4.67e-20, 1.50e-15, 7.45e-19 and 2.99e-11, respectively, Table 2). In the multivariate Cox analysis, only age, grade and TACC3 expression were independent risk factors for OS (p < 0.001, p = 0.004 and p < 0.001, respectively). These findings indicated that TACC3 expression was an independent risk factor for OS among KIRC patients (HR = 1.12, 95% CI: 1.06-1.18, p < 0.001, Figure 3).

**Table 2:** Univariate and multivariate analysis of the correlation of TACC3 expression with OS among KIRC patients.

**Parameter**	**Univariate analysis**			**Multivariate analysis**		
	**HR**	**95% CI**	**p-value**	**HR**	**95% CI**	**p-value**
Age	1.03	1.02-1.05	2.29e-06***	1.04	1.02-1.05	<0.001***
Gender	0.93	0.68-1.28	0.66	1.01	0.73-1.41	0.95
Grade	2.32	1.87-2.87	9.48e-15***	1.43	1.12-1.83	0.004**
Stage	1.89	1.65-2.16	4.67e-20***	1.54	0.99-2.38	0.05
T classification	1.94	1.64-2.30	1.50e-15***	0.91	0.61-1.35	0.65
M classification	4.28	3.11-5.91	7.45e-19***	1.49	0.76-2.91	0.24
N classification	0.86	0.74-1.01	0.07	0.84	0.71-0.99	0.033*
TACC3	2.35	1.83-3.02	2.99e-11***	1.12	1.06-1.18	<0.001***

* Values indicate p<0.05, ** values indicate p<0.01, *** values indicate p<0.001.

### Association between TACC3 expression and tumor immune infiltration in KIRC

**Figure 4:**
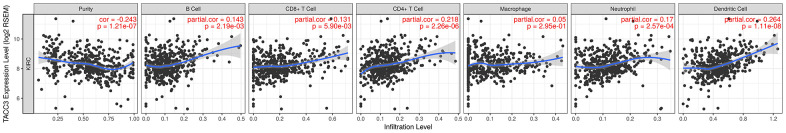
**Association between TACC3 expression and tumor immune cell infiltration (purity, B cell, CD8+ T cell, CD4+ T cell, macrophage, neutrophil and DCs) in KIRC.** The data were determined using TIMER.

Recently, tumor-infiltrating lymphocytes have been shown to play an important role in mediating response to chemotherapy and improving clinical outcomes in various cancer types, especially in KIRC. Here, TIMER-based analysis was used to determine the correlation between TACC3 expression and levels of tumor infiltrating immune cells in KIRC. Interestingly, high TACC3 expression correlated with poor prognosis and high infiltration levels (Figure 4). TACC3 expression positively correlated with infiltrating B cells (partial. cor = 0.143, p = 2.19e-03), CD8+ T cell (partial. cor = 0.131, p = 5.90e-03), CD4+ T cells (partial. cor = 0.218, p=2.26e-06), neutrophils (partial. cor = 0.17, p = 2.57e-04), and dendritic cells (DCs, partial. cor = 0.264, p = 1.11e-08) in KIRC tissues. In addition, TACC3 expression demonstrated a significant negative correlation with tumor purity in KIRC (cor = -0.243, p = 1.21e-07).

To further confirm these results, RNA-seq data for KIRC were downloaded from TCGA database and tumor infiltration levels for 22 immune cell subtypes were subsequently analyzed by CIBERSORT. We compared TACC3 expression with the estimated abundances of these 22 immune cell types by Spearman correlation analysis (Table 3). In addition to the four immune cell types (B cells, CD8+ T cell, CD4+ T cells, and DCs) observed by TIMER, CIBERSORT demonstrated that TACC3 expression in KIRC tissues significantly correlated with several other types of immune cells, including follicular helper T cells, regulatory T (Tregs) cells, gamma delta T cells, resting natural killer (NK) cells, monocytes, activated DCs, and resting mast cells. These results suggest that TACC3 may be closely associated with immune cell infiltration in KIRC.

**Table 3:** Correlation of TACC3 expression and 22 types of immune cells in KIRC using CIBERSORT.

**Immune cells**	**r**	**95% CI**	**p-value**
B cells naive	-0.193	-0.2885 to -0.09380	0.0001***
B cells memory	0.122	0.02161 to 0.2207	0.0144*
Plasma cells	0.094	-0.007111 to 0.1932	0.0606
T cells CD8	0.249	0.1513 to 0.3410	<0.0001****
T cells CD4 naive	-	-	-
T cells CD4 memory resting	-0.247	-0.3398 to -0.1499	<0.0001****
T cells CD4 memory activated	0.216	0.1177 to 0.3104	<0.0001****
T cells follicular helper	0.248	0.1508 to 0.3406	<0.0001****
T cells regulatory (Tregs)	0.309	0.2142 to 0.3972	<0.0001****
T cells gamma delta	0.134	0.03382 to 0.2323	0.0072**
NK cells resting	-0.168	-0.2647 to -0.06829	0.0007***
NK cells activated	-0.056	-0.1560 to 0.04551	0.2661
Monocytes	-0.114	-0.2121 to -0.01258	0.0234*
Macrophages M0	-0.018	-0.1189 to 0.08316	0.7192
Macrophages M1	0.028	-0.07314 to 0.1288	0.5754
Macrophages M2	-0.174	-0.2705 to -0.07449	0.0005
Dendritic cells resting	0.060	-0.04135 to 0.1600	0.2321
Dendritic cells activated	-0.221	-0.3153 to -0.1230	<0.0001****
Mast cells resting	-0.278	-0.3689 to -0.1823	<0.0001****
Mast cells activated	-0.049	-0.1495 to 0.05209	0.3267
Eosinophils	-0.010	-0.1110 to 0.09110	0.8414
Neutrophils	-0.006	-0.1069 to 0.09522	0.9065

* Values indicate p<0.05, ** values indicate p<0.01, *** values indicate p<0.001, **** values indicate p<0.0001. – Data equal zero. Spearman correlation was used in the study.

### Confirmation of the correlation between TACC3 expression and immune infiltration-associated markers

**Figure 5:**
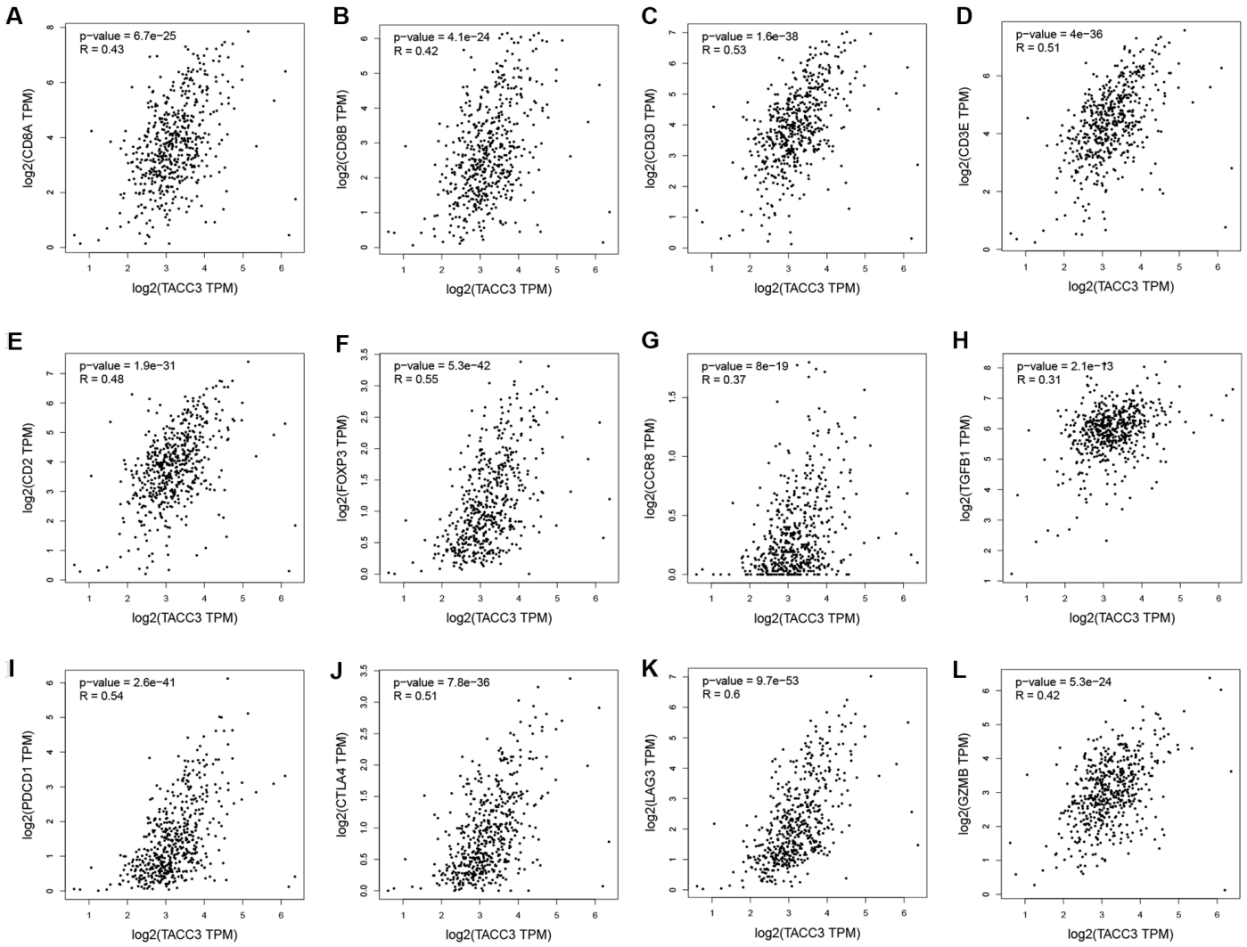
**Correlation between TACC3 expression and immune infiltration-associated marker genes.** CD8+ T cells: CD8A (**A**) and CD8B (**B**). T cells (general): CD3D, CD3E and CD2 (**C**–**E**). Treg: FOXP3, CCR8 and TGFB1 (**F**–**H**). T cell-exhaustion: PD-1, CTLA4, LAG3 and GZMB (**I**–**L**).

To confirm the relationship between TACC3 expression and immune cell infiltration levels in KIRC, GEPIA and UALCAN were used to determine the correlations between TACC3 expression and immune infiltration associated markers [21] (Table 4 and Figure 5). Our analysis revealed that TACC3 expression significantly correlated with 88.1% (37/42) of the immune markers identified in KIRC, which validated the results of our previous analyses and further suggested that TACC3 plays a significant role in tumor immune infiltration (p < 0.05). Additionally, we determined the relationship between TACC3 expression and T cell exhaustion-associated markers, including PD-1, CTLA4, LAG3, TIM-3 and GZMB. Our results revealed that there was a significant correlation between the expression of TACC3 and PD-1, CTLA4, LAG3 and GZMB (r = 0.42-0.60, p < 0.001, Figure 5). Significant correlations were also observed between TACC3 expression and the Treg associated markers FOXP3, CCR8, STAT5B and TGFβ. These results indicate that TACC3 expression levels are significantly associated with multiple immune markers in KIRC.

**Table 4:** Correlation analysis between TACC3 and immune infiltration associated markers.

**Immune cells**	**Markers**	**Tumor**^1^		**Normal**^1^		**Tumor**^2^
		**R**	**P**	**R**	**P**	**Pearson-CC**
CD8+ T cell	CD8A	0.43	***	0.13	0.29	0.46
	CD8B	0.42	***	-0.02	0.87	0.55
T cell (general)	CD3D	0.51	***	0.21	0.079	0.38
	CD3E	0.51	***	0.094	0.43	0.38
	CD2	0.48	***	0.07	0.56	0.85
B cell	CD19	0.48	***	0.12	0.32	0.38
	CD79A	0.38	***	-0.039	0.75	0.42
Monocyte	CD86	0.33	***	0.17	0.15	0.66
	CD115 (CSF1R)	0.42	***	0.098	0.41	0.55
TAM	CCL2	0.11	*	0.5	***	0.42
	CD68	0.14	***	-0.15	0.2	0.82
	IL10	0.26	***	0.26	*	0.45
M1 Macrophage	INOS (NOS2)	0.075	0.088	0.055	0.65	0.37
	IRF5	0.37	***	0.74	***	0.37
	COX2 (PTGS2)	0.076	0.083	0.39	***	0.81
M2 Macrophage	CD163	0.33	***	0.21	0.071	0.37
	VSIG4	0.33	***	0.2	0.097	-0.3
	MS4A4A	0.24	***	0.07	0.56	0.43
Neutrophils	CD66b (CEACAM8)	0.028	0.53	0.1	0.38	0.32
	CD11b (ITGAM)	0.38	***	0.013	0.91	0.61
	CCR7	0.38	***	0.17	0.17	0.81
Natural killer cell	KIR2DL1	0.11	*	-0.11	0.36	0.6
	KIR2DL3	0.14	***	-0.069	0.56	0.59
	KIR2DL4	0.3	***	-0.035	0.77	0.68
	KIR3DL1	0.065	0.14	-0.00094	0.99	0.31
	KIR3DL2	0.17	***	-0.16	0.19	0.35
	KIR3DL3	0.11	*	0.12	0.33	0.3
	KIR2DS4	0.13	**	-0.13	0.26	0.44
Dendritic cell	HLA-DPB1	0.35	***	0.41	***	0.45
	HLA-DQB1	0.27	***	0.21	0.079	0.75
	HLA-DRA	0.28	***	0.43	***	0.39
	HLA-DPA1	0.26	***	0.37	**	0.55
	BDCA-1 (CD1C)	0.19	***	0.049	0.69	0.46
Dendritic cell	BDCA-4 (NRP1)	-0.063	0.15	-0.036	0.76	-
	CD11c (ITGAX)	0.52	***	0.08	0.51	0.66
Tfh	BCL6	0.11	**	0.75	***	0.53
	IL21	0.17	***	0.15	0.21	0.46
Treg	FOXP3	0.55	***	0.43	***	0.39
	CCR8	0.37	***	0.074	0.54	0.41
	STAT5B	-0.095	*	0.19	0.11	0.38
	TGFβ (TGFB1)	0.31	***	0.82	***	-0.3
T cell exhaustion	PD-1	0.54	***	-0.067	0.58	0.3
	CTLA4	0.51	***	0.12	0.32	0.45
	LAG3	0.6	***	0.72	***	0.68
	TIM-3 (HAVCR2)	0.061	0.16	-0.47	***	-0.3
	GZMB	0.42	***	0.054	0.65	0.38
	PDL1	0.11	*	0.59	***	-

1 Indicated data determined by GEPIA; 2 indicated data determined by UALCAN.

Abbreviations: TAM, tumor-associated macrophage; Tfh, Follicular helper T cell; Treg, regulatory T cell.

- Not mentioned, * Values indicated p<0.05, ** values indicated p<0.01, *** values indicated p<0.001.

### Tumor immune infiltration in low and high TACC3-expressing KIRC tissues

**Figure 6:**
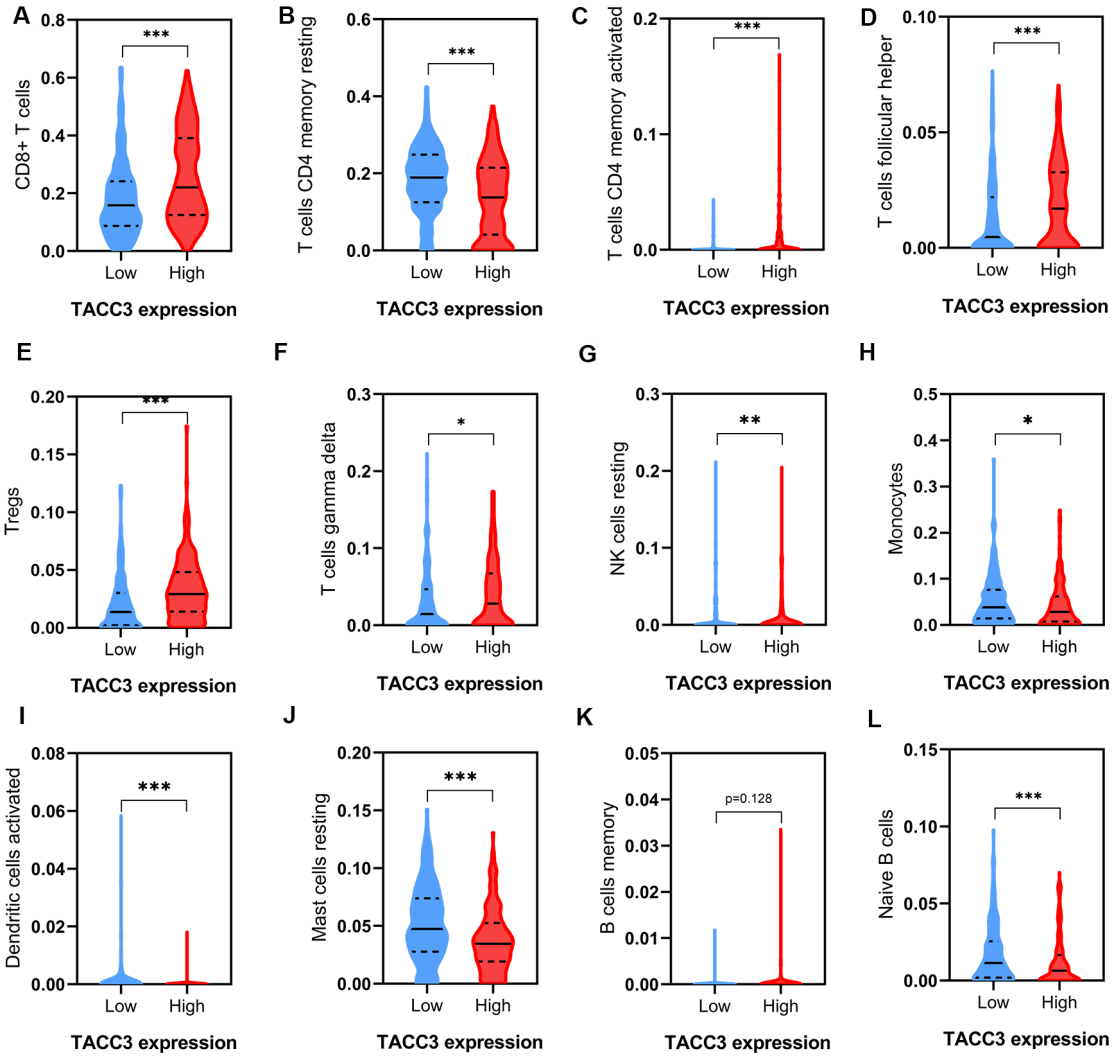
**Tumor immune infiltrates in high and low TACC3-expressing KIRC tissues.** (**A**) CD8+ T cells. (**B**, **C**) CD 4+ T cells. (**D**) T follicular helper cells. (**E**) Tregs. (**F**) Gamma delta T cells. (**G**) Resting NK cells. (**H**) Monocytes. (**I**) Activated Dendritic cells. (**J**) Resting Mast cells. (**K**, **L**) B cells. The blue and red violins represent immune infiltrated cells in low and high TACC3 expressing groups, respectively. The solid lines represent median expressions of infiltrated cells and the dotted lines represent quartiles. * p<0.05, ** p<0.01, *** p<0.001.

To further elucidate the intrinsic relationship between tumor infiltrating immune cells and TACC3 expression in KIRC, we detected the expression signatures of 12 immune cell subtypes in low and high TACC3-expressing tumors. Expression signatures for 91.67% (11/12) of the infiltrating immune cell subtypes were identified in the low and high TACC3 expression groups (Figure 6). Interestingly, the expression signatures for six types of infiltrating cells, included CD8+ T cells, CD4+ memory T cells, follicular helper T cells, Tregs and resting NK cells were higher in the high TACC3 group, when compared with the low TACC3 group. The expression signatures for five types of infiltrating cells, which included T cells, resting CD4 memory cells, monocytes, activated DCs, resting mast cells and naive B cells, were higher in the low TACC3 group. These results suggest that TACC3 may be associated with various subtypes of tumor-infiltrating immune cells in KIRC.

### Correlations between TACC3 expression and T cell exhaustion-associated markers

**Figure 7:**
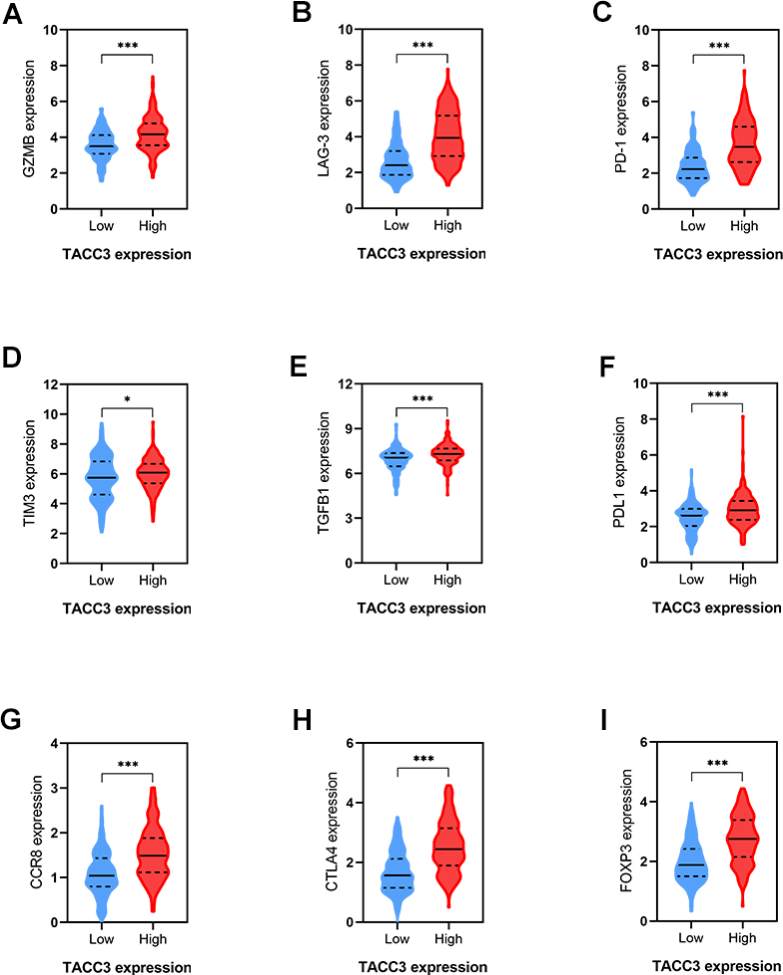
**Correlations between T cells exhaustion-associated markers and TACC3 expression.** (**A**) GZMB, (**B**) LAG-3, (**C**) PD-1, (**D**) TM3, (**E**) TGFβ1, (**F**) PDL1, (**G**) CCR8, (**H**) CTLA4, (**I**) FOXP3. The blue and red violins represent T cells-exhaustion associated markers in low and high TACC3 expressing groups, respectively. All these markers showed significant differences between TACC3 high and low expressing groups. The solid lines represent median expressions of infiltrated cells and the dotted lines represent quartiles. * p<0.05, ** p<0.01, *** p<0.001.

As shown above (Table 4 and Figure 5), TACC3 may be associated with T cell exhaustion. We therefore further examined the association between TACC3 expression and the expression of the T cell exhaustion-associated markers PD-1, CTLA4, PDL1, LAG3, GZMB, TIM3, TGFβ1, CCR8, and FOXP3. All these markers were more highly expressed in the high TACC3 expression group, when compared with the low TACC3 expression group (Figure 7). These results strongly suggest that TACC3 expression is associated with T cell exhaustion markers.

### High expression of TACC3 mRNA in KIRC tissues

**Figure 8:**
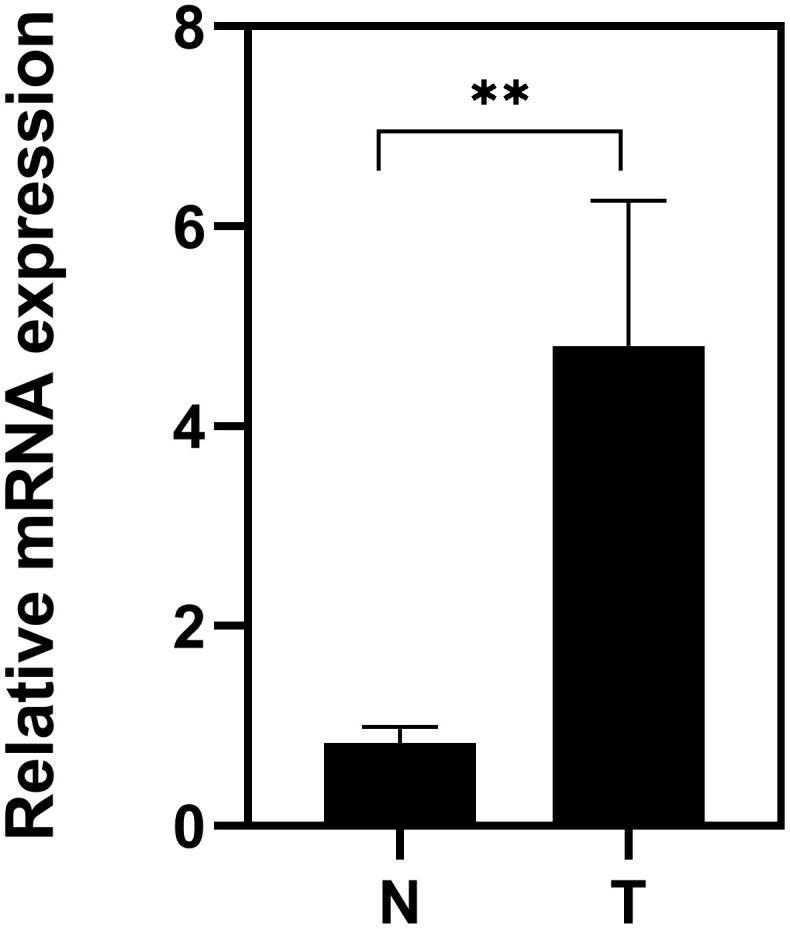
**Expression of TACC3 mRNA in KIRC tissues.** T: KIRC tissues; N: noncancerous tissues. The results were shown with Mean±SD. N=10, **p<0.01.

To further confirm the TACC3 mRNA expression in KIRC tissues, we performed Real-Time PCR in 10 pairs of matched KIRC tissues and their noncancerous tissues. As shown in Figure 8, the expression of TACC3 mRNA was overexpressed in KIRC tissues, compared with the noncancerous tissues (p<0.01).

### KEGG pathways analysis

**Figure 9:**
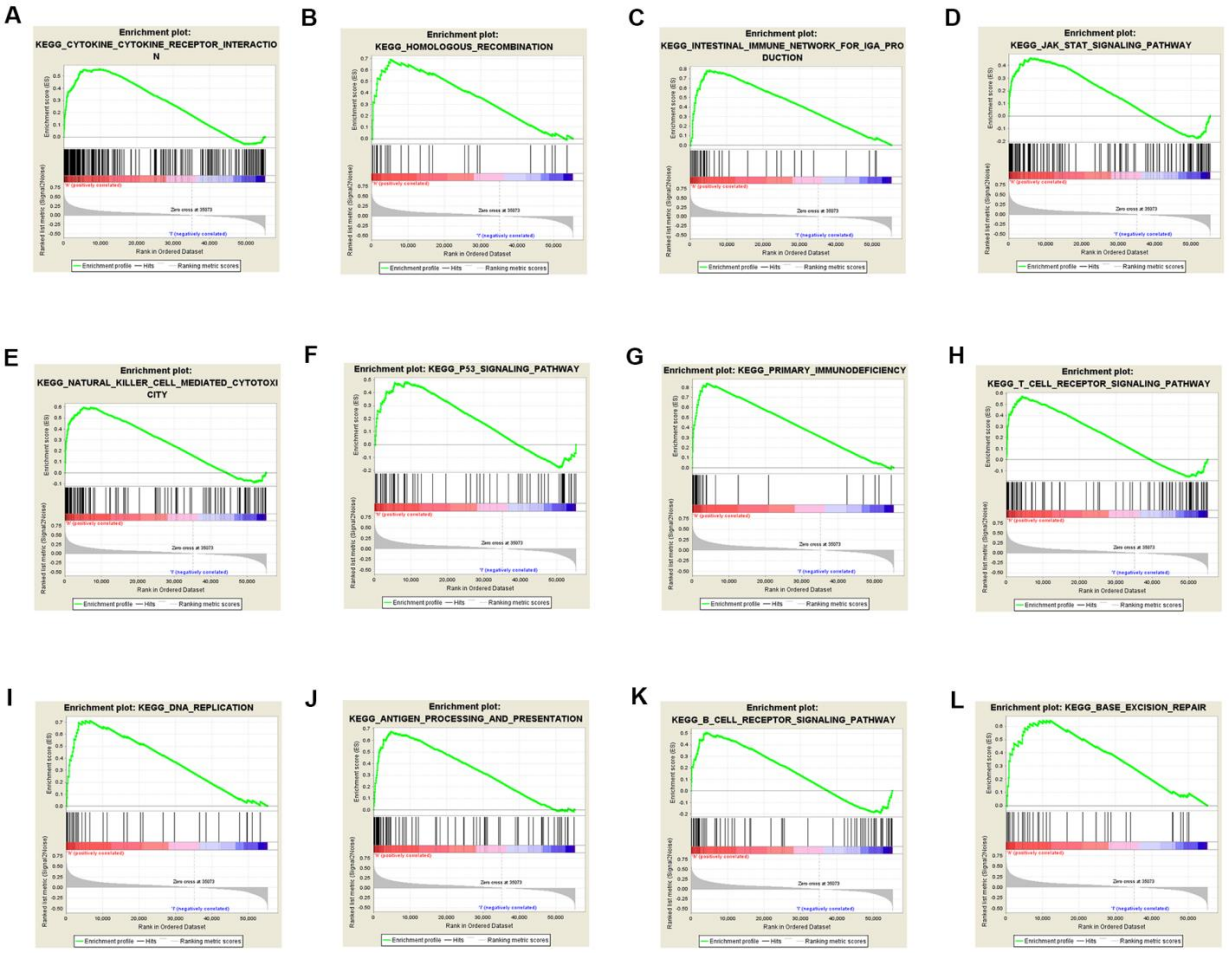
**Gene sets enriched in the high TACC3 expression phenotype using GSEA.** The results showing immune-associated pathways: cytokine-cytokine receptor interaction (**A**), intestinal immune network for IgA production (**C**), natural killer cell-mediated cytotoxicity (**E**), primary immunodeficiency (**G**), T cell receptor signaling (**H**), antigen processing and presentation (**J**) and B cell receptor signaling (**K**); p53 signaling pathway (**F**); JAK/STAT signaling pathway (**D**); DNA repair-associated pathways: homologous recombination (**B**), DNA replication (**I**), and base excision repair (**L**).

To identify TACC3 associated signaling pathways in KIRC, we performed KEGG analysis and compared the high and low TACC3 expression datasets. The analysis revealed that 87/178 gene sets are upregulated in the high TACC3 expression phenotype, and 91/178 gene sets are upregulated in the low TACC3 expression phenotype. Gene sets differentially enriched in the high TACC3 expression phenotype included many genes related to immunomodulation, such as autoimmune thyroid disease, cytokine-cytokine receptor interaction, primary immunodeficiency, NK cell-mediated cytotoxicity, and antigen processing and presentation (Table 5 and Figure 9). Conversely, the low TACC3 expression phenotype showed enrichment of a series of metabolic gene sets, including propanoate metabolism, pyruvate metabolism, proximal tubule bicarbonate reclamation and the citric acid cycle (Table 6).

**Table 5:** Gene sets enriched in the high TACC3 expression phenotype.

**Gene set name**	**NES**	**NOM p-value**	**FDR q-value**
KEGG_AUTOIMMUNE_THYROID_DISEASE	2.395	0	5.24E-04
KEGG_CYTOKINE_CYTOKINE_RECEPTOR_INTERACTION	2.365	0	5.32E-04
KEGG_INTESTINAL_IMMUNE_NETWORK_FOR_IGA_PRODUCTION	2.278	0	0.002
KEGG_PRIMARY_IMMUNODEFICIENCY	2.279	0	0.002
KEGG_NATURAL_KILLER_CELL_MEDIATED_CYTOTOXICITY	2.209	0	0.004
KEGG_ASTHMA	2.212	0	0.004
KEGG_VIRAL_MYOCARDITIS	2.188	0	0.004
KEGG_ANTIGEN_PROCESSING_AND_PRESENTATION	2.152	0	0.007
KEGG_ALLOGRAFT_REJECTION	2.128	0	0.008
KEGG_HOMOLOGOUS_RECOMBINATION	2.036	0	0.014
KEGG_GRAFT_VERSUS_HOST_DISEASE	2.013	0.002	0.014
KEGG_CYTOSOLIC_DNA_SENSING_PATHWAY	2.047	0.002	0.013
KEGG_TYPE_I_DIABETES_MELLITUS	2.090	0.004	0.010
KEGG_HEMATOPOIETIC_CELL_LINEAGE	2.103	0.004	0.009
KEGG_LEISHMANIA_INFECTION	2.069	0.006	0.011
KEGG_CELL_ADHESION_MOLECULES_CAMS	2.077	0.006	0.011
KEGG_AMYOTROPHIC_LATERAL_SCLEROSIS_ALS	1.749	0.006	0.063
KEGG_FC_GAMMA_R_MEDIATED_PHAGOCYTOSIS	1.977	0.006	0.018
KEGG_CHEMOKINE_SIGNALING_PATHWAY	2.008	0.008	0.014
KEGG_BASE_EXCISION_REPAIR	2.029	0.008	0.014
KEGG_GLYCOSAMINOGLYCAN_BIOSYNTHESIS_CHONDROITIN_SULFATE	1.850	0.008	0.039
KEGG_JAK_STAT_SIGNALING_PATHWAY	1.917	0.010	0.025
KEGG_DNA_REPLICATION	1.924	0.012	0.027
KEGG_VEGF_SIGNALING_PATHWAY	1.720	0.014	0.068
KEGG_T_CELL_RECEPTOR_SIGNALING_PATHWAY	2.023	0.015	0.013
KEGG_SYSTEMIC_LUPUS_ERYTHEMATOSUS	1.858	0.020	0.038
KEGG_ALPHA_LINOLENIC_ACID_METABOLISM	1.561	0.024	0.133
KEGG_FC_EPSILON_RI_SIGNALING_PATHWAY	1.738	0.026	0.065
KEGG_PROTEASOME	1.770	0.027	0.062
KEGG_NOD_LIKE_RECEPTOR_SIGNALING_PATHWAY	1.769	0.027	0.060
KEGG_NEUROACTIVE_LIGAND_RECEPTOR_INTERACTION	1.497	0.031	0.160
KEGG_CELL_CYCLE	1.923	0.031	0.026
KEGG_P53_SIGNALING_PATHWAY	1.725	0.037	0.068
KEGG_B_CELL_RECEPTOR_SIGNALING_PATHWAY	1.752	0.044	0.064
KEGG_TOLL_LIKE_RECEPTOR_SIGNALING_PATHWAY	1.698	0.047	0.074

Abbreviations: NES, normalized enrichment score; NOM, nominal; FDR, false discovery rate. Gene sets with NOM p-value <0.05 and FDR q-value <0.25 were considered as significantly enriched.

**Table 6:** Gene sets enriched in the low TACC3 expression phenotype.

**Gene set name**	**NES**	**NOM p-value**	**FDR q-value**
KEGG_PROPANOATE_METABOLISM	-2.284	0	0.006
KEGG_PYRUVATE_METABOLISM	-2.321	0	0.009
KEGG_PROXIMAL_TUBULE_BICARBONATE_RECLAMATION	-2.037	0	0.055
KEGG_BUTANOATE_METABOLISM	-1.998	0.004	0.053
KEGG_CITRATE_CYCLE_TCA_CYCLE	-2.021	0.004	0.051
KEGG_VALINE_LEUCINE_AND_ISOLEUCINE_DEGRADATION	-2.082	0.006	0.048
KEGG_REGULATION_OF_AUTOPHAGY	-1.747	0.008	0.108
KEGG_ALDOSTERONE_REGULATED_SODIUM_REABSORPTION	-1.796	0.010	0.102
KEGG_GLYCOSYLPHOSPHATIDYLINOSITOL_GPI_ANCHOR_BIOSYNTHESIS	-1.845	0.010	0.101
KEGG_TIGHT_JUNCTION	-1.904	0.010	0.084
KEGG_VASOPRESSIN_REGULATED_WATER_REABSORPTION	-1.898	0.010	0.077
KEGG_FATTY_ACID_METABOLISM	-1.922	0.012	0.082
KEGG_TRYPTOPHAN_METABOLISM	-1.829	0.014	0.094
KEGG_THYROID_CANCER	-1.796	0.014	0.095
KEGG_GLYCOLYSIS_GLUCONEOGENESIS	-1.786	0.016	0.095
KEGG_SPHINGOLIPID_METABOLISM	-1.687	0.023	0.115
KEGG_LONG_TERM_POTENTIATION	-1.628	0.024	0.138
KEGG_TGF_BETA_SIGNALING_PATHWAY	-1.770	0.027	0.099
KEGG_TYPE_II_DIABETES_MELLITUS	-1.621	0.028	0.133
KEGG_ENDOMETRIAL_CANCER	-1.802	0.030	0.105
KEGG_BIOSYNTHESIS_OF_UNSATURATED_FATTY_ACIDS	-1.692	0.031	0.122
KEGG_INSULIN_SIGNALING_PATHWAY	-1.725	0.032	0.112
KEGG_PEROXISOME	-1.839	0.033	0.096
KEGG_ADIPOCYTOKINE_SIGNALING_PATHWAY	-1.703	0.035	0.120
KEGG_PPAR_SIGNALING_PATHWAY	-1.640	0.036	0.135
KEGG_TERPENOID_BACKBONE_BIOSYNTHESIS	-1.692	0.037	0.117
KEGG_ETHER_LIPID_METABOLISM	-1.496	0.038	0.167
KEGG_RENIN_ANGIOTENSIN_SYSTEM	-1.601	0.047	0.129
KEGG_MELANOMA	-1.500	0.047	0.169
KEGG_ADHERENS_JUNCTION	-1.726	0.050	0.117

## DISCUSSION

TACC3 plays an important role in regulating cell mitosis, transcription, and tumorigenesis. However, the expression pattern and roles of TACC3 in renal cell carcinoma still remain unclear. There is increasing evidence to suggest that TACC3 is associated with various types of human cancers, including breast [12, 22, 23], prostate [13], colorectal [14], bladder [24], gastric [15], ovarian [25], lung [26], melanoma [27], and liver cancer [28]. Moreover, FGFR3-TACC3 gene fusions have been reported to be a common chromosomal abnormality across all tumor types [18, 19]. Together, these studies suggest that TACC3 may play an important role in tumor progression. In this study, we examined the expression and prognostic value of TACC3 in KIRC. In order to eliminate any discrepancies in levels of TACC3 expression and prognostic potential in KIRC, a variety of databases, including Oncomine, TIMER, Kaplan-Meier plotter, GEPIA, UALCAN, and TCGA, were analyzed. Our analysis revealed a consistent correlation between abnormally high TACC3 expression and poor patient prognosis in KIRC, suggesting that TACC3 expression may be a valuable prognostic biomarker for this disease (Figures 1, 2). Consistent with this, univariate and multivariate Cox analyses indicated that TACC3 mRNA expression may be a useful biomarker for KIRC cancer prognosis (Figure 3). Together, these data strongly suggest that TACC3 is a prognostic biomarker in KIRC. In recent years, immunotherapy has been clinically validated as an effective treatment option for many tumors such as melanoma, non-small cell lung cancer and renal cell carcinoma, suggesting that immune cell infiltration may play a significant role in KIRC formation and therapy [4, 26, 29, 30]. Therefore, we determined the role of TACC3 in immune infiltration in KIRC. The results obtained from our TIMER and CIBERSORT analysis demonstrated that there was a significant association between the level of TACC3 expression and the degree of tumor infiltration by B cells, CD8+ T cells, CD4+ T cells and DCs in KIRC tissues. In contrast, none of the methods found any significant association between macrophage infiltration (p > 0.05) and TACC3 expression level (Figure 4 and Table 3). In addition, our CIBERSORT analysis also identified a strong association between TACC3 expression and tumor infiltration by follicular helper T cells, Tregs, gamma delta T cells, resting NK cells, resting mast cells and monocytes (Table 3). To further confirm these findings, correlations between TACC3 expression and immune cell associated marker genes were determined by GEPIA and UALCAN. This independent analysis also demonstrated a strong association between TACC3 expression and many of the same immune cell subtypes identified in the previous analysis (Table 4). In addition, a significant correlation in the expression of TACC3 with a number of T cell exhaustion markers (LAG3, PD-1, CTLA4, TIM-3) was also observed (Figure 5). Notably, one more intriguing finding is that high TACC3 expression correlates with high infiltration levels of immune cells. Till now, there are no reports regarding the direct relationship between TACC3 and immune cells. However, some studies suggest that TACC3 may be indirectly involved in the progress of T cell lineage [31, 32]. NF-κB signaling is known as a major regulator for T lineage cells [33]. TACC3 overexpression can promote NF-κB signaling to help T cell development at different stages [34]. In addition, TACC3 expression can regulate T cell differentiation via PI3K/AKT signaling [16]. Therefore, TACC3-induced NF-κB and/or PI3K/AKT signaling may be involved in different stage of T cell development. However, the exact mechanisms still need to be investigated. Taken together, these results suggest that TACC3 correlates with immune cell infiltrations in KRIC.

Because tumor-infiltrating lymphocytes correlate with improved prognosis in many cancers [35–37], we determined the levels of immune cell infiltrating in low and high TACC3-expressing tissues using data from TCGA. Significant differences were observed for most of the tumor-infiltrating lymphocytes examined (Figure 6A–6L). Interestingly, high CD8+ T cell infiltration was found in tissues with high TACC3 expression (Figure 6A), which is associated with poor OS in KIRC (Figure 2F). This observation seems paradoxical given the assumption that the presence of CD8+ T cells would be predictive of patient survival [29, 38]. However, T cell exhaustion has been extensively described as a mechanism for suppressing the ability of CD8+ T cells to proliferate and kill tumor cells [37, 39]. Indeed, we found that high expression of the T cell exhaustion markers PD-1, CTLA4, LAG-3, GZMB, and TIM3, as well as high expression of the Treg markers FOXP3, CCR8 and TGFβ1, were all associated with high TACC3 expression, supporting the idea that T cell exhaustion could suppress T cell functions within these tumors (Figure 7). Thus, these findings could explain why the high level of CD8+ T cells could not induce a survival benefit in KIRC. Recently, Han et al. have reported that T-cell receptor diversity in peripheral blood PD1+ CD8+ T cells could predict clinical outcomes in patients with non-small cell lung cancer, suggesting that levels of CD8+ T cells together with T cell exhaustion markers may serve as useful clinical biomarkers in cancer [40]. In the present study, we demonstrate that TACC3 is closely associated with tumor immune infiltration and T cell exhaustion in KIRC. However, future studies are needed to elucidate the intrinsic mechanisms by which TACC3 regulates this process.

Findings from an increasing number of studies provide possible mechanistic explanations for the relationship between high TACC3 expression and poor prognosis in various cancer types. Recently, Ha et al. have reported that TACC3 can promote an epithelial-mesenchymal transition (EMT) phenotype through activation of PI3K/AKT and ERK signaling pathways in cervical cancer [16]. Mounting evidence indicates that EMT phenotypes are accompanied with (1) up-regulation of mesenchymal markers, such as N-cadherin, vimentin and MMP-9 [41, 42] and (2) down-regulation of the epithelial marker E-cadherin [16]. TACC3-mediated induction of EMT in human cervical cancer cells could be explained as follows: overexpressed TACC3 may increase the expression of Snail and Slug, as well as the transcriptional activity of β-catenin, by (1) increasing the phosphorylation of AKT and ERK1/2 and/or (2) decreasing GSK3β activation, a downstream target of the PI3K/AKT signaling pathway. Conversely, TACC3 knockdown inactivates PI3K/AKT signaling in KIRC cells, suggesting that PI3K/AKT signaling may be involved in tumor growth [20]. In the current study, our GSEA results showed that TACC3 was associated with JAK/STAT signaling pathway (NOM p = 0.010, FDR q = 0.025, Table 5), which is known to regulate cell growth, survival and differentiation. Disrupted or dysregulated JAK/STAT function can result in immune deficiency syndromes and cancers. In addition, phosphorylated JAK can activate STAT, as well as PI3K/AKT. Thus, a possible mechanism may be that knockdown of TACC3 inactivates PI3K/AKT by suppressing the JAK/STAT pathway.

In the present study, we have confirmed that TACC3 is strongly associated with tumor immune infiltration. To further understand the underlying mechanisms of TACC3 in KIRC, KEGG analysis was performed using GSEA. This analysis revealed that TACC3 was strongly associated with various types of immune-related pathways (Figure 8). Our current results confirm that TACC3 is associated with immune infiltration in KIRC. In addition, Lai et al. have reported that the hallmarks of KIRC formation include diverse signaling pathways, such as p53 signaling, immune destruction avoidance, and DNA repair [4]. Our GSEA results confirmed that these pathways were all enriched among the high TACC3 expression phenotypes. However, our study still has certain limitations as follows: 1) more clinical data for KIRC patients should be analyzed to judge the relationship between TACC3 expression and patient prognosis; 2) future research is required to explore the detailed mechanism between TACC3 expression and immune cell infiltration/T cell exhaustion; 3) as TACC3 may be a suitable biomarker for KIRC, TACC3-targeted drugs should be explored in the future.

In summary, based upon our bioinformatics analysis from Oncomine and TCGA databases, we found that high TACC3 expression is associated with poor patient prognosis of KIRC and we further found that this phenotype is also associated with increased immune cell infiltration and T cell exhaustion. Therefore, TACC3 may represent a novel biomarker of patient prognosis in KIRC.

## MATERIALS AND METHODS

### Oncomine database

The Oncomine database is a powerful set of analysis functions that compute gene expression signatures, clusters and gene-set modules, automatically extracting insightful biological information from 715 datasets and 86, 733 samples (https://www.oncomine.org/resource/login.html) [43]. In this study, the expression levels of TACC3 in various types of tumors was determined by Oncomine database analysis. The thresholds (p≤0.0001, fold change: 1.5, and gene rank: all) were considered as statistically significant.

### TCGA database analysis

Gene expression data and patient data for KIRC were downloaded from the Genomic Data Commons (GDC) data portal (https://portal.gdc.cancer.gov/) using the GDC data transfer tool. Gene expression data were analyzed using R statistical software (version: 3.6.1) with related R packages. Clinical parameters, such as age, gender, survival, and tumor grade and stage were extracted from the patient data and then matched to each patient using a PERL script.

### GEPIA database analysis

The Gene Expression Profiling Interactive Analysis (GEPIA) platform (http://gepia.cancer-pku.cn/) is a newly developed interactive web server for analyzing RNA sequencing expression data for 9, 736 tumors and 8, 587 normal samples from The Cancer Genome Atlas (TCGA) and the Genotype-Tissue Expression database projects, using a standard processing pipeline [44]. These databases were used to evaluate TACC3 expression in various cancer types. ANOVA was used for the comparison of tumor tissues with paired normal tissues, with the following thresholds: |log_2_|FC cutoff = 1 and q-value cutoff = 0.01. In the survival analysis, the threshold was determined according to the following values: group cutoff: median; cutoff-high (%): 50; cutoff-low (%): 50. In the correlation analysis, the correlation coefficient was determined using the Spearman method. The TCGA tumor and normal tissue datasets were used for further analysis.

### UALCAN database analysis

UALCAN is a comprehensive and interactive web resource (http://ualcan.path.uab.edu/index.html) for analyzing cancer OMICS data [45]. UALCAN is designed to provide easy access to publicly available cancer OMICS data (from the TCGA and MET500 databases), allowing users to identify biomarkers of interest. In this study, TACC3 expression data was analyzed from TCGA databases and p < 0.05 was considered statistically significant.

### Kaplan-Meier plotter database analysis

Based on a meta-analysis, the Kaplan Meier plotter (http://kmplot.com/analysis/) is capable of evaluating the effect of 54, 000 genes on survival in 21 cancer types [46]. The correlation between TACC3 and survival in KIRC was analyzed using RNA-seq data. The patients were divided into low and high expression groups according to median expression, and the cutoff value was set to ‘auto select’.

### TIMER database analysis

The Tumor IMmune Estimation Resource (TIMER) is a comprehensive resource for the systematical analysis of immune infiltrates across 32 cancer types (https://cistrome.shinyapps.io/timer/) [47]. TIMER employs a novel statistical method to estimate the abundances of six tumor infiltrating immune cell types (B cells, CD4+ T cells, CD8+ T cells, neutrophils, macrophages and dendritic cells (DCs)) and has been validated using pathological estimations [48]. In this study, correlations between TACC3 expression and the above six immune cell types were determined by Spearman’s correlation analysis.

### CIBERSORT analysis

CIBERSORT, a computational approach developed by Newman et al, aims to characterize the cell composition of complex tissues from their gene expression profiles [49]. In the current study, CIBERSORT was performed using an R script (version:3.6.1) to determine the abundances of 22 tumor-infiltrating immune cell subsets in KIRC.

### KEGG analysis

To identify the potential signaling mechanisms underlying the effects of TACC3 expression on KIRC prognosis, GSEA was performed to detect whether a priori defined set of genes showed statistically significant differential expression between the high and low TACC3 expression groups [50]. Gene sets with a normal p value < 0.05 and false discovery rate (FDR) < 0.05 were considered significantly enriched.

### KIRC patient tissues

Fresh KIRC tissues from cases that were histologically confirmed and did not undergo any other treatments were obtained from The Fourth Hospital of Hebei Medical University. The study was approved by the Institute Research Ethics Committee at The Fourth Hospital of Hebei Medical University.

### RNA extraction and real-time PCR

Real-Time PCR was performed to determine the expression of TACC3 in 10 KIRC patients at RNA levels. Briefly, total RNA from the surgically obtained paired tissues was isolated using TRI Reagent RNA Isolation Reagent (Sigma-Aldrich) according to the manufacturer's instructions. A reverse transcription system was used to obtain cDNA. The PCR reaction was described as follows: (a) 94° C for 3 minutes; (b) 35 cycles of 94° C for 30 seconds, 56° C for 30 seconds, 72° C for 2 minutes; and (c) 72° C for 10 minutes. The primers are described as follows: TACC3: 5'-CCTCTTCAAGCGTTTTGAGAAAC-3' (sense) and 5'-GCCCTCCTGGGTGATCCTT-3' (antisense); β-actin: 5'-CGCGAG AAGATGACCCAGAT-3' (sense) and 5'-GGGCATACCCCT CGTAGATG-3' (antisense). The relative fold change in each sample was calculated using the 2^-ΔΔCt^ method normalized to β-actin. Each sample was performed in triplicate.

### Statistical analysis

Most of analyses were conducted using R software (version 3.6.1). Univariate Cox analysis was used to select potential prognostic factors, and multivariate Cox analysis was performed to verify the correlations between TACC3 expression and survival, along with other clinical features. Some statistical tests were performed with IBM SPSS Statistics 26. Two-tailed p values less than 0.05 were considered statistically significant.

## Supplementary Material

Supplementary Table 1
