# Supplementary material for: TACC3 is a prognostic biomarker for kidney renal clear cell carcinoma and correlates with immune cell infiltration and T cell exhaustion
Source: Aging (Albany NY). 2021 Mar 10;13(6):8541–62. doi: 10.18632/aging.202668 (PMC8034911; doi:10.18632/aging.202668)
Supplement: Supplementary Table 1 [file aging-13-202668-s001.pdf]

SUPPLEMENTARY TABLE

Supplementary Table 1. The relationship between TACC3 overexpression and patient prognosis (Overall Survival) in different cancers.

Tumor types	TACC3 Overexpression	p-value
ACC	-	0.25
BLCA	+	0.55
BRCA	+	0.25
CESC	+	0.41
CHOL	-	0.12
COAD	+	0.15
DLBC	+	0.17
ESCA	-	0.39
GBM	+	0.36
HNSC	+	0.52
KICH	-	0.09
**KIRC**	+	**0.00003*** **
KIRP	-	0.0019**
LAML	-	0.0094**
LGG	-	0.00000000078***
**LIHC**	+	**0.0028** **
LUAD	-	0.0001**
LUSC	+	0.36
MESO	-	0.00003***
OV	+	0.65
PAAD	+	0.27
PCPG	-	0.36
PRAD	-	0.11
READ	+	0.86
SARC	-	0.71
SKCM	+	0.52
STAD	+	0.24
TGCT	-	0.93
THCA	-	0.77
**THYM**	+	**0.013* **
UCEC	+	0.96
UCS	+	0.089
UVM	-	0.13

The extension of these abbreviations was shown below; – means TACC not overexpression, + means TACC overexpression; P values mean patient overall survival between low and high TACC3. *p<0.05, **p<0.01, ***p<0.001.

Notes:

Tumor types:

ACC	Adrenocortical carcinoma
BLCA	Bladder Urothelial Carcinoma
BRCA	Breast invasive carcinoma
CESC	Cervical squamous cell carcinoma and endocervical adenocarcinoma
CHOL	Cholangio carcinoma
COAD	Colon adenocarcinoma
DLBC	Lymphoid Neoplasm Diffuse Large B-cell Lymphoma
ESCA	Esophageal carcinoma
GBM	Glioblastoma multiforme
HNSC	Head and Neck squamous cell carcinoma
KICH	Kidney Chromophobe
KIRC	Kidney renal clear cell carcinoma
KIRP	Kidney renal papillary cell carcinoma
LAML	Acute Myeloid Leukemia
LGG	Brain Lower Grade Glioma
LIHC	Liver hepatocellular carcinoma
LUAD	Lung adenocarcinoma
LUSC	Lung squamous cell carcinoma
MESO	Mesothelioma
OV	Ovarian serous cystadenocarcinoma
PAAD	Pancreatic adenocarcinoma
PCPG	Pheochromocytoma and Paraganglioma
PRAD	Prostate adenocarcinoma
READ	Rectum adenocarcinoma
SARC	Sarcoma
SKCM	Skin Cutaneous Melanoma
STAD	Stomach adenocarcinoma
TGCT	Testicular Germ Cell Tumors
THCA	Thyroid carcinoma
THYM	Thymoma
UCEC	Uterine Corpus Endometrial Carcinoma
UCS	Uterine Carcinosarcoma
UVM	Uveal Melanoma
